# Intravenous Tranexamic Acid Reduces Perioperative Blood Loss in Reduction Mammoplasty With Immediate Implant-Based Reconstruction: A Randomized, Triple-Blinded, Placebo-Controlled Trial

**DOI:** 10.1093/asj/sjaf258

**Published:** 2025-12-09

**Authors:** Jerzy Kolasiński, Tomasz Reysner, Małgorzata Kolenda, Szymon Kołacz, Małgorzata Reysner

## Abstract

**Background:**

Postoperative hematoma and oozing can compromise outcomes after reduction mammoplasty with immediate reconstruction. Intravenous (IV) tranexamic acid (TXA) is antifibrinolytic, but prospective evidence in this setting is limited.

**Objectives:**

The aim of this study was to determine whether a single pre-incision dose of IV TXA reduces perioperative blood loss and fibrinolytic activation vs placebo.

**Methods:**

In this randomized, triple-blinded, placebo-controlled trial, 60 women (American Society of Anesthesiologists I/II, 18-75 years) undergoing bilateral reduction mammoplasty with immediate implant-based reconstruction received TXA 10 mg/kg in 100 mL saline or placebo 10 min before incision. The primary outcome was total blood loss within 24 h (intraoperative suction + swab plus drain output). Secondary outcomes were perioperative changes in hemoglobin, D-dimer and fibrinogen, and complications within 30 days. Intention-to-treat analyses were performed.

**Results:**

All patients completed follow-up. Total blood loss was lower with TXA than with placebo (mean ± standard deviation: 221.1 ± 72.4 vs 298.1 ± 90.6 mL; mean difference −77.0 mL; 95% CI, −122.4 to −31.6; *P* = .001). Intraoperative loss and 24 h drain output were also reduced. Postoperative D-dimer rise was attenuated with TXA (0.31 ± 0.15 vs 0.49 ± 0.22 µg/mL; *P* = .002); hemoglobin decline was smaller. No thromboembolic, neurologic, or allergic events occurred; no skin-flap necrosis was observed.

**Conclusions:**

Pre-incisional IV TXA safely reduces perioperative bleeding and fibrinolytic activity after reduction mammoplasty. These findings support incorporation of IV TXA into perioperative protocols.

**Level of Evidence: 2 (Therapeutic):**

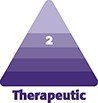

In aesthetic and reconstructive plastic surgery, perioperative blood loss and postoperative hematoma formation continue to pose challenges that can affect surgical results and patient satisfaction.^1^ Although breast aesthetic procedures such as augmentation, reduction, or mastopexy involve relatively moderate intraoperative bleeding, even small postoperative collections can distort delicate tissue contours, delay recovery, and increase the need for secondary interventions.^2^ Meticulous hemostasis and drain placement are therefore critical; however, pharmacologic adjuncts capable of minimizing diffuse capillary oozing are increasingly of clinical interest.^3^

Tranexamic acid (TXA), a synthetic lysine analog, is an antifibrinolytic agent that competitively inhibits the activation of plasminogen to plasmin, thereby stabilizing fibrin clots and reducing fibrinolysis at the surgical wound.^4^ TXA has been widely investigated in orthopedic, trauma, and cardiovascular surgery, where it has consistently demonstrated efficacy in reducing perioperative blood loss and transfusion requirements without significantly increasing the risk of thromboembolism.^5-7^ In recent years, attention has shifted toward its use in soft-tissue and aesthetic plastic surgery, where controlling bleeding and achieving aesthetic precision are significant.^6^

Accumulating evidence supports the use of TXA in procedures such as abdominoplasty, rhinoplasty, and facelift surgery, demonstrating significant reductions in intraoperative blood loss, postoperative drainage, and ecchymosis without compromising safety.^8-10^ Recent systematic reviews and meta-analyses have also confirmed that both systemic and topical administration of TXA are associated with lower rates of postoperative hematoma and improved early recovery in patients undergoing plastic surgery.^10,11^ Despite these findings, evidence from breast aesthetic surgery remains limited, and most available data are derived from retrospective or heterogeneous cohorts combining reconstructive and cosmetic cases.

Breast surgery presents a unique bleeding profile.^2^ The surgical field involves highly vascularized glandular and subcutaneous tissue, where diffuse capillary oozing can persist despite meticulous electrocautery.^10^ In this setting, TXA's antifibrinolytic action could play a valuable role in promoting stable clot formation and reducing serosanguinous drainage.^12^ However, because baseline blood loss is usually lower than in large-volume body-contouring procedures, the extent and clinical importance of TXA's effect in purely aesthetic breast surgery need further clarification.

The current randomized, triple-blind, placebo-controlled clinical trial was designed to assess the efficacy and safety of a single pre-incisional intravenous dose of TXA (10 mg/kg) in women undergoing breast aesthetic surgery. The primary goal was to determine whether TXA administration decreases total perioperative blood loss compared with placebo. Secondary goals included evaluating its effects on perioperative hemoglobin decline, fibrinolytic activity reflected by D-dimer and fibrinogen levels, and the occurrence of postoperative complications. We hypothesized that a single intravenous dose of TXA given before incision would significantly decrease perioperative blood loss and fibrinolytic activation compared with placebo, without raising adverse events. We prospectively assessed whether a single pre-incisional intravenous dose of TXA (10 mg/kg) reduces total 24 h perioperative blood loss in women undergoing reduction mammoplasty. We hypothesized that TXA would lower blood loss and fibrinolytic activation (D-dimer) relative to placebo without increasing adverse events.

## METHODS

### Study Design and Ethics

This prospective, randomized, triple-blinded, placebo-controlled clinical trial was conducted in Klinika Kolasiński and at Poznan University of Medical Sciences (Poznań, Poland) between September 2023 and September 2025. The study protocol was approved by the Bioethical Commission at Poznan University of Medical Sciences (approval no. 544/2023) and registered on ClinicalTrials.gov (NCT05945680). All participants provided written informed consent before enrollment. The study adhered to the principles outlined in the Declaration of Helsinki and the guidelines of Good Clinical Practice.

### Participants

Eligible participants were adult women aged 18 to 75 years scheduled for elective reduction mammoplasty. Only patients classified as American Society of Anesthesiologists (ASA) physical status I or II were included. Patients with ASA >II were excluded to ensure a homogeneous, low-risk population and to minimize confounding from systemic comorbidities that could affect coagulation and perioperative bleeding. Obesity (BMI > 35 kg/m^2^) was also an exclusion criterion, as it is associated with altered fibrinolysis and increased thromboembolic risk, which could bias the assessment of TXA safety and efficacy. Although the trial was broadly registered for breast aesthetic surgery, the enrolled cohort consisted of patients undergoing bilateral mastectomy with immediate implant-based reconstruction. All included patients had reduction mammaplasty for both health and aesthetic reasons. Typical intraoperative blood loss in purely aesthetic breast augmentation procedures at our institution is minimal (<50 mL) and moderate for mastopexy (100-200 mL), whereas reduction mammoplasty generally results in higher volumes (∼250-300 mL). These reference values show that the current cohort represents the higher end of bleeding in aesthetic breast surgery. This change reflected the patient population routinely treated at our institution during the study period and did not alter the original study design, intervention, or outcome measures. Eligibility criteria remained consistent with the registered protocol (ASA I/II, BMI 20-35 kg/m^2^, age 18-75 years, and absence of thromboembolic or hematologic disorders). Indications for reduction mamoplasty included: physical (medical) symptoms such as chronic neck pain, shoulder and upper back pain, recurrent skin infections beneath the breast, postural problems, headaches, neuropathy or paresthesia in the upper limbs, functional problems, and psychological complaints caused by poor body image or low self-esteem. Exclusion criteria were: BMI <20 or >35 kg/m^2^; ASA physical status of III or higher; history of thromboembolic disease; known hematological disorders; ongoing chronic anticoagulant or antiplatelet therapy (such as aspirin within the past 14 days or anticoagulants within the past 5 days before surgery); epilepsy; hypersensitivity to TXA; or any known coagulation abnormalities.

### Randomization and Blinding

Randomization and allocation concealment were rigorously carried out to reduce bias. Eligible participants were assigned in a 1:1 ratio to either receive intravenous TXA (TXA group) or a placebo (control group). The randomization sequence was generated using dedicated statistical software (GraphPad Prism, version 10.5.0; GraphPad Software, San Diego, CA) with a variable block size of 4 to 6 to ensure balanced allocation and prevent prediction of group assignment. This block randomization method helped maintain nearly equal group sizes even with a modest total sample and minimized potential selection bias. The 1:1 allocation ratio was maintained throughout enrollment until the target of 60 patients was reached. A study coordinator, not involved in patient care, data collection, or outcome assessment, prepared the randomization list. Each allocation code was sealed in a sequentially numbered, opaque, sealed envelope, which was only opened after the participant was enrolled and deemed eligible. To maintain methodological integrity, the trial was conducted using a triple-blind design. Neither the participants, the surgical and anesthesia teams, nor the investigators who performed postoperative assessments and data analyses were aware of the assigned interventions. A pharmacist not involved in the trial prepared the study medications. Both TXA and placebo solutions were prepared in identical 100 mL syringes containing either TXA (10 mg/kg diluted in 0.9% normal saline) or an equal volume of normal saline. The syringes appeared identical and were labeled only with the participant's study identification number. This process ensured blinding was maintained throughout the perioperative and analytical phases, preventing any potential influence of treatment knowledge on surgical procedures, postoperative care, or data interpretation.

### Interventions

All participants received the assigned intervention according to a standardized perioperative protocol. Patients assigned to the TXA group were given an intravenous dose of TXA at 10 mg/kg of body weight, diluted in 100 mL of 0.9% sodium chloride solution. The infusion was administered slowly over 5 to 10 min and finished at least 10 min before the skin incision. This timing was chosen to ensure adequate plasma levels of the antifibrinolytic agent during the period of maximum surgical bleeding. Patients in the control group received an identical volume of 0.9% normal saline over the same duration and at the same time before incision. The preparation and administration of both TXA and placebo solutions were performed by a nurse anesthetist who was not involved in any other part of the study, ensuring blinding of the surgical and anesthesia teams. Anesthetic management was carefully standardized to reduce potential confounding effects on intraoperative hemodynamics and bleeding. General anesthesia was induced with intravenous propofol at ∼2 mg/kg and fentanyl at 2 µg/kg, followed by endotracheal intubation facilitated with rocuronium. Anesthesia was maintained using sevoflurane in a 50:50 mixture of oxygen and medical air, with continuous monitoring of capnography, electrocardiography, and pulse oximetry. Mean arterial pressure, heart rate, and oxygen saturation were recorded periodically throughout the procedure. Additional doses of fentanyl were titrated as needed to maintain hemodynamic stability and prevent sympathetic responses to surgical stimulation. All surgical procedures were performed by the same senior plastic surgeon and team to ensure procedural consistency.

The surgical technique, including the method of incision, dissection plane, and extent of tissue undermining, was a standardized inverted T reduction mammoplasty with supermedial pedicle flap used for all patients. Hemostasis was carefully achieved using bipolar electrocautery, and closed-suction drains were routinely inserted in all cases. Drains were evaluated every 8 to 12 h and removed after 24 to 48 h once output was <30 mL per drain. No significant difference in the timing of drain removal was observed between groups. Wounds were closed in multiple layers with absorbable sutures. Postoperative management was also standardized, including the use of a compression garment, drain care, and monitoring for hematoma formation. This high level of procedural and anesthetic uniformity was designed to minimize variability and enable a reliable assessment of the effect of TXA on perioperative blood loss and fibrinolytic activity.

### Outcome Measures

The primary outcome of the study was the total perioperative blood loss, measured in milliliters as the sum of intraoperative and postoperative losses within the first 24 h after surgery. Intraoperative blood loss was calculated by subtracting the volume of irrigation solution used from the total volume of fluid collected in the suction canisters. Additionally, all surgical swabs were weighed before and after use, and the weight difference was converted to milliliters of blood using the standard ratio of 1 g equals 1 mL. Postoperative blood loss was determined by recording the total drainage output collected in the closed-suction drains during the first 24 h after surgery. All measurements were performed by an anesthesiology nurse blinded to group allocation, following a predefined protocol to ensure accuracy and consistency. The total blood loss for each participant was obtained by adding the intraoperative and postoperative volumes, providing an accurate estimate of perioperative hemorrhage.

Secondary outcomes included laboratory and safety parameters that provided insights into the physiological effects of TXA and the overall safety profile of the intervention. Hemoglobin concentration, expressed in grams per deciliter, was measured in venous blood samples collected immediately before anesthesia induction and again 24 h after surgery. The perioperative change in hemoglobin served as an indirect indicator of blood loss and hemodilution. D-dimer levels, reported in micrograms per milliliter, were analyzed in the same time frame to assess fibrinolytic activity and clot stability. The concentration of fibrinogen, expressed in milligrams per deciliter, was also determined preoperatively and 24 h postoperatively to evaluate the impact of TXA on coagulation dynamics. All blood samples were obtained through peripheral venipuncture and processed in the hospital's central clinical laboratory. D-dimer levels were measured using an automated immunoturbidimetric assay, and fibrinogen concentration was determined by the Clauss method. Laboratory personnel were blinded to treatment assignment to prevent any potential bias in sample handling or interpretation.

Safety outcomes were continuously monitored throughout hospitalization and during the 30-day postoperative period. Special attention was given to detecting thromboembolic events, including deep vein thrombosis and pulmonary embolism, as well as seizures, allergic or hypersensitivity reactions, wound hematomas, and any reoperations or unplanned readmissions. Clinical evaluations were conducted daily during the inpatient stay, and all adverse events were documented and verified by the attending surgical team. This comprehensive outcome assessment aimed to evaluate both the effectiveness of TXA in reducing perioperative blood loss and its safety profile in patients undergoing breast aesthetic surgery.

### Sample Size Calculation

The sample size was determined prospectively, with the primary endpoint defined as total perioperative blood loss (in milliliters) within 24 h after surgery. Estimations were based on preliminary institutional data collected during the pretrial audit period (June-August 2023) at our center. In this internal dataset, 20 patients who underwent bilateral reduction mammoplasty with immediate aesthetic reconstruction, without TXA, had an average total blood loss of ∼290 ± 95 mL. The internal dataset was used solely for sample size estimation and did not serve as an additional control group in this trial. The term aesthetic reconstruction refers to immediate breast reconstruction following mastectomy using prosthetic implants and aesthetic shaping techniques, specifically skin-sparing mastectomy with direct-to-implant reconstruction. This terminology was chosen to highlight the aesthetic design aspects of reconstruction rather than oncologic parameters. In comparison, a pilot group of 10 patients who received a single pre-incision intravenous dose of TXA showed a mean total blood loss of ∼210 ± 85 mL. This indicated a between-group difference of roughly 70 to 80 mL, with a pooled standard deviation (SD) of ∼90 to 95 mL.

Assuming a conservative mean difference of 70 mL and an SD of 95 mL, with a 2-tailed significance level (*α*) of .05 and a statistical power (1 – *β*) of .80, the minimum required sample size was calculated to be 26 participants per group. To account for potential dropouts, missing data, or protocol deviations, the sample size was increased to 30 participants per group, totaling 60 participants. This sample size was considered sufficient to detect a clinically meaningful reduction in blood loss, corresponding to ∼0.5 to 0.7 g/dL less postoperative hemoglobin decline, and a decreased risk of hematoma formation or prolonged drain output. The study was not specifically powered for secondary biochemical outcomes such as D-dimer or fibrinogen levels, which were regarded as exploratory endpoints. No interim analysis or sample size re-estimation was planned. All calculations were performed using GraphPad StatMate 2.0 (GraphPad Software).

### Statistical Analysis

All statistical analyses were conducted on an intention-to-treat basis, including every randomized patient in the final analysis according to their original group assignment. Data integrity was confirmed before analysis, and all calculations were performed using GraphPad Prism, version 10.5.0 (GraphPad Software). GraphPad Prism was chosen for its validated biomedical statistical modules, reproducibility in previous surgical randomized controlled trials, and compliance with CONSORT and ICMJE reporting standards. Continuous variables were tested for normality using the Shapiro–Wilk test and visually examined with histograms and Q–Q plots. Variables following a normal distribution were presented as mean ± SD, whereas non-normally distributed variables were reported as medians with interquartile ranges. Categorical data were expressed as counts and percentages.

Comparisons between the TXA and control groups were performed using appropriate parametric or nonparametric tests based on data distribution. Specifically, Student's *t* test for independent samples was used for normally distributed variables (eg, age, BMI, blood loss, and laboratory parameters). At the same time, the Mann–Whitney *U* test was applied to non-normal variables. Categorical variables, such as ASA physical status and postoperative complication rates, were compared with the *χ*^2^ test or Fisher's exact test, depending on expected cell counts.

Within-group comparisons of preoperative and postoperative laboratory values (hemoglobin, D-dimer, and fibrinogen) were analyzed using paired *t* tests for normally distributed data or Wilcoxon signed-rank tests for nonparametric data. The change magnitude (Δ) within each group was computed as the difference between postoperative and preoperative values and reported as mean ± SD.

For each comparison between groups, mean differences were calculated and presented with 95% CI to assess precision and clinical relevance. A *P*-value of <.05 was deemed statistically significant. No correction for multiple testing was made, as all secondary outcomes were exploratory and aimed to support the interpretation of the primary endpoint.

Data completeness was evaluated before analysis, and no missing outcome data were identified. The dataset contained no extreme outliers warranting exclusion. For sensitivity analysis, the primary outcome (total blood loss) was also examined after logarithmic transformation to verify the normality assumptions; results remained consistent, so untransformed mean values are reported for clarity.

The statistical plan was formulated before unblinding and strictly followed the prespecified protocol. The primary analysis compared total perioperative blood loss between groups. Secondary analyses included intraoperative and drain losses, perioperative changes in hemoglobin, D-dimer, and fibrinogen levels, as well as postoperative complication rates. All results are reported with exact *P*-values to facilitate interpretation of both statistical and clinical significance.

## RESULTS

### Participant Characteristics

**Figure 1.**
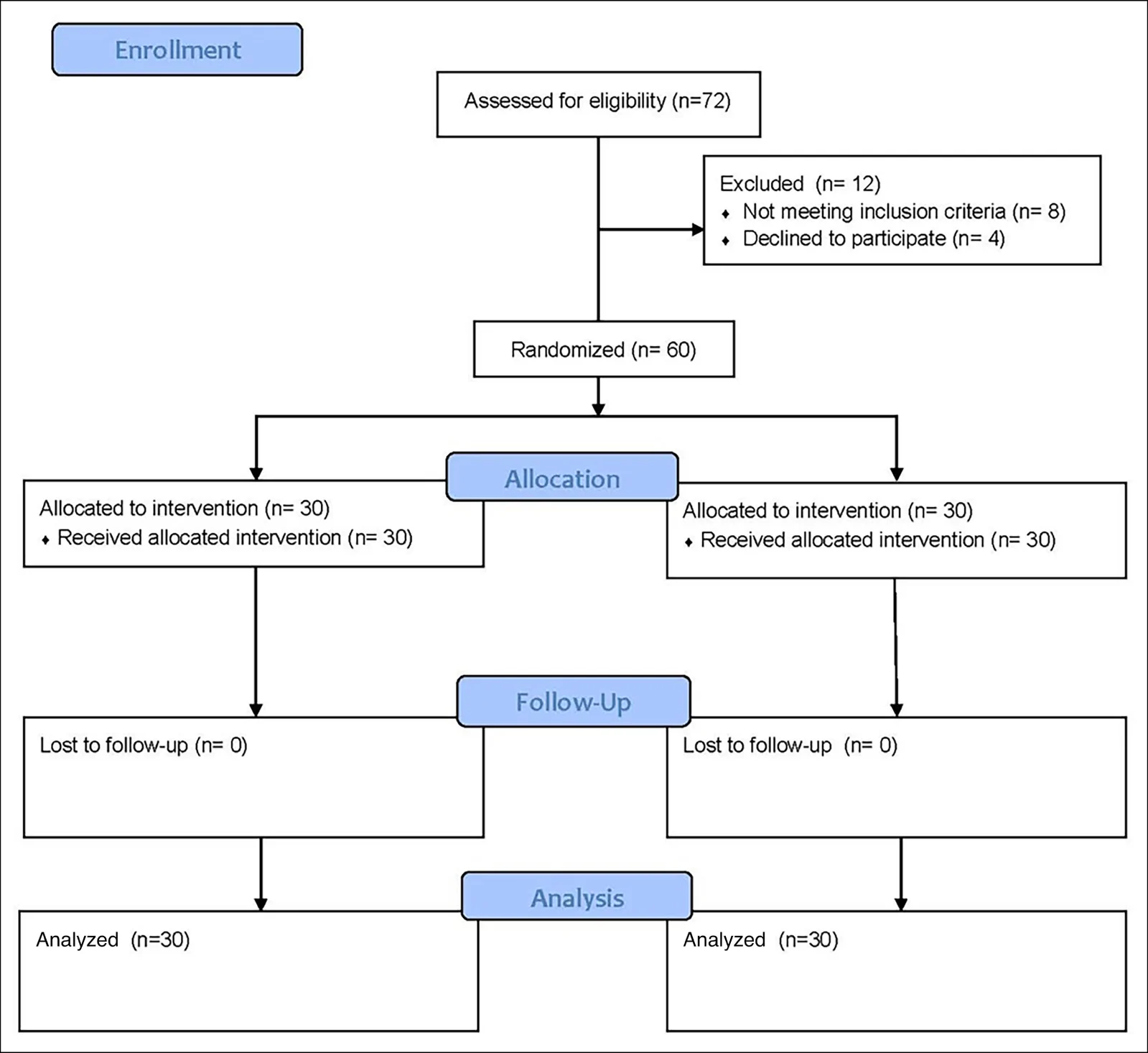
CONSORT flow diagram of patient enrollment, randomization, and analysis.

Between September 2023 and September 2025, a total of 72 patients were screened for eligibility. Twelve patients were excluded: 8 did not meet the inclusion criteria (BMI > 35 kg/m^2^ or chronic anticoagulant therapy) and 4 declined to participate. The remaining 60 patients met all inclusion criteria and were randomized to receive either intravenous TXA (TXA group, *n* = 30) or placebo (control group, *n* = 30). All randomized patients received their assigned intervention and completed both the 24 h postoperative evaluation and the 30-day safety follow-up. There were no protocol deviations or losses to follow-up. The participant flow through the study is presented in Figure 1.

Baseline demographic and clinical characteristics were comparable between groups (Table 1). The mean age was 42.8 ± 8.1 years in the TXA group and 43.2 ± 7.6 years in the control group (*P* = .839). The enrolled patients’ age range was 27 to 58 years. The groups were similar with respect to BMI (24.9 ± 3.8 vs 25.4 ± 4.0 kg/m^2^, *P* = .633), weight, height, and ASA physical status distribution (ASA I/II ratio 24/6 vs 23/7, *P* = .79). No statistically significant differences were found for any baseline parameter, confirming adequate randomization and group comparability.

**Table 1.** Baseline Demographic and Clinical Characteristics of Study Participants

Variable	TXA (*n* = 30) Mean ± SD	Control (*n* = 30) Mean ± SD	Mean difference	95% CI for difference	*P*-value^b^
Age (years)	42.8 ± 8.1	43.2 ± 7.6	−0.4	−4.3 to 3.5	.839
BMI (kg/m^2^)	24.9 ± 3.8	25.4 ± 4.0	−0.5	−2.6 to 1.6	.633
Weight (kg)^a^	67.2 ± 8.9	68.3 ± 10.2	−1.1	−6.4 to 4.2	.678
Height (cm)^a^	165.8 ± 6.2	166.4 ± 7.0	−0.6	−4.0 to 2.8	.715
ASA I/II ratio	24/6	23/7	—	—	.79

ASA, American Society of Anesthesiologists physical status classification; SD, standard deviation; TXA, tranexamic acid. ^a^Weight and height were extracted from operative records (rounded values; descriptive only). ^b^Compared using Fisher's exact test. No statistically significant differences were observed between groups at baseline.

Comorbidities were minimal and evenly distributed between groups. The most common were mild controlled hypertension (3 patients in the TXA group and 2 in the control), hypothyroidism on stable levothyroxine therapy (2 vs 2), and well-managed anxiety disorders (1 vs 1). No patients had diabetes, cardiovascular disease, or coagulation disorders. A detailed overview of baseline demographic and clinical characteristics, including comorbidities, is provided in Table 1.

### Primary and Secondary Outcomes

**Figure 2.**
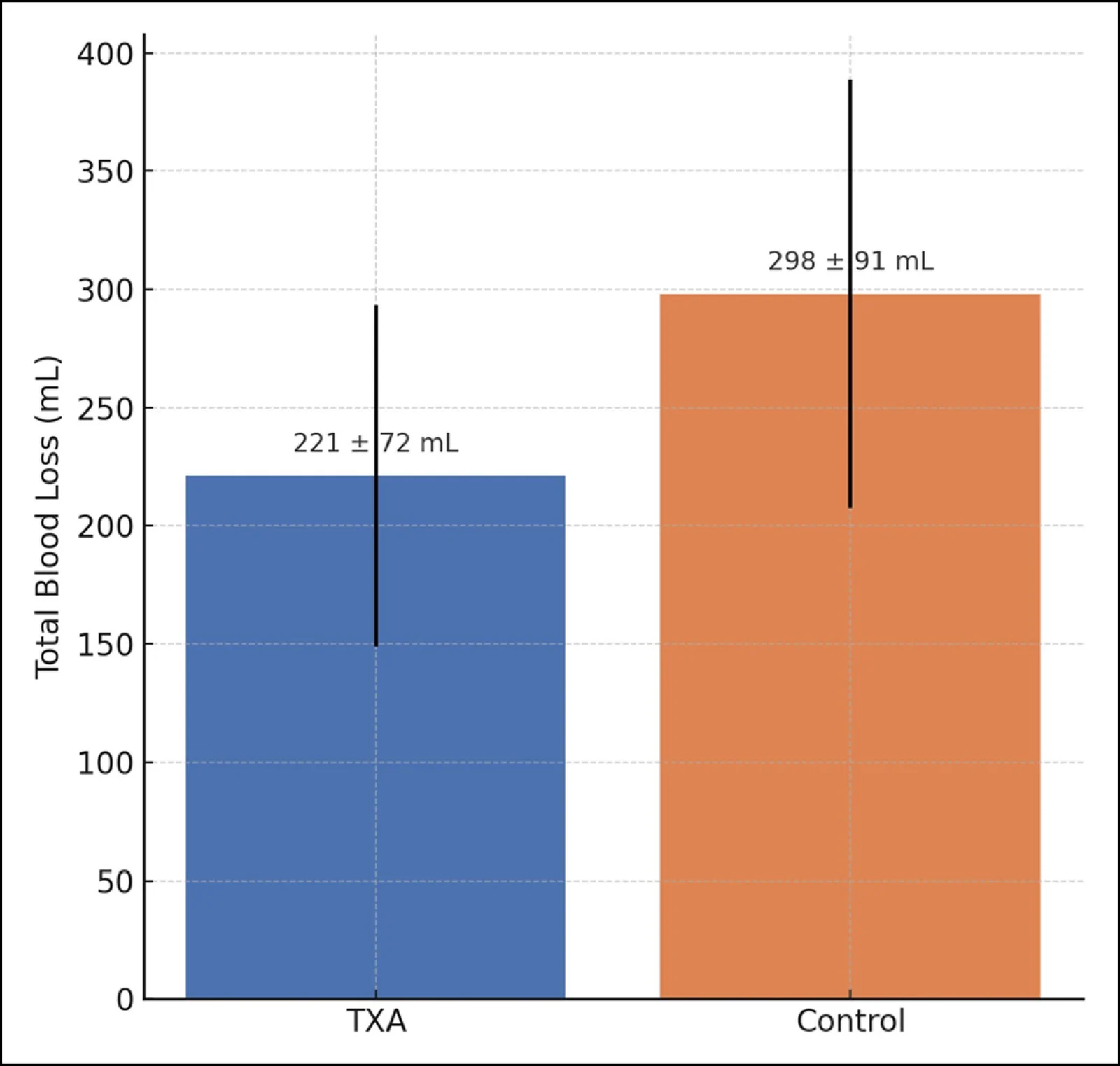
Primary outcome: total perioperative blood loss within 24 h.

TXA administration significantly reduced perioperative blood loss compared with placebo (Table 2, Figure 2). The mean intraoperative blood loss was 92.4 ± 38.5 mL in the TXA group and 121.6 ± 49.3 mL in the control group (mean difference: −29.2 mL; 95% CI, −52.8 to −5.6; *P* = .017). Similarly, the 24 h drain output was lower in the TXA group (128.7 ± 64.2 mL) compared with controls (176.5 ± 79.6 mL), yielding a mean difference of −47.8 mL (95% CI, −86.3 to −9.3; *P* = .016). When combined, the total perioperative blood loss was 221.1 ± 72.4 mL in patients receiving TXA vs 298.1 ± 90.6 mL in the control group—a reduction of 77.0 mL (95% CI, −122.4 to −31.6; *P* = .001). A post hoc power analysis for the primary endpoint confirmed 83% power to detect the observed between-group difference of 77 mL (*α* = .05). Intraoperative blood loss and 24 h drain output were also significantly reduced in the TXA group. The total perioperative fluid balance, including intraoperative infusion and postoperative drainage, was similar between the groups.

**Table 2.** Intraoperative, Postoperative, and Total Blood Loss Within 24 h After Surgery

Variable	TXA (*n* = 30) Mean ± SD	Control (*n* = 30) Mean ± SD	Mean difference	95% CI for difference	*P*-value
Intraoperative blood loss (mL)	92.4 ± 38.5	121.6 ± 49.3	−29.2	−52.8 to −5.6	.017
Drain output (mL, 24 h)	128.7 ± 64.2	176.5 ± 79.6	−47.8	−86.3 to −9.3	.016
Total blood loss (mL, 24 h)	221.1 ± 72.4	298.1 ± 90.6	−77.0	−122.4 to −31.6	.001
Hemoglobin pre-op (g/dL)	13.24 ± 1.12	13.36 ± 1.15	−0.12	−0.73 to 0.49	.695
Hemoglobin 24 h (g/dL)	12.78 ± 1.18	12.33 ± 1.25	0.45	−0.22 to 1.12	.181
D-dimer pre-op (µg/mL)	0.27 ± 0.13	0.26 ± 0.12	0.01	−0.06 to 0.09	.734
D-dimer 24 h (µg/mL)	0.31 ± 0.15	0.49 ± 0.22	−0.18	−0.28 to −0.08	.002
Fibrinogen pre-op (mg/dL)	2.90 ± 0.75	2.94 ± 0.71	−0.04	−0.45 to 0.37	.840
Fibrinogen 24 h (mg/dL)	3.08 ± 0.70	3.44 ± 0.85	−0.36	−0.74 to 0.02	.064

SD, standard deviation; TXA, tranexamic acid.

Hemoglobin concentration decreased postoperatively in both groups but to a lesser extent in patients who received TXA. The mean postoperative hemoglobin was 12.78 ± 1.18 g/dL in the TXA group and 12.33 ± 1.25 g/dL in controls (*P* = .181). Within-group analysis confirmed that hemoglobin fell by −0.46 ± 0.40 g/dL in the TXA group (*P* = .001) compared with −1.03 ± 0.49 g/dL in the control group (*P* < .001), indicating a smaller decline in the TXA cohort (Table 3). With respect to fibrinolytic markers, D-dimer levels remained stable in the TXA group (0.27 ± 0.13 µg/mL preoperatively vs 0.31 ± 0.15 µg/mL at 24 h, *P* = .132). In contrast, a marked increase was observed in controls (0.26 ± 0.12 µg/mL vs 0.49 ± 0.22 µg/mL, *P* < .001). Between-group comparison of postoperative D-dimer levels revealed a statistically significant difference (−0.18 µg/mL; 95% CI, −0.28 to −0.08; *P* = .002), suggesting attenuation of fibrinolysis following TXA administration.

**Table 3.** Perioperative Laboratory Parameters: Hemoglobin, D-dimer, and Fibrinogen Levels

Variable	TXA pre-op Mean ± SD	TXA 24 h Mean ± SD	Δ Mean ± SD	*P*-value	Control pre-op Mean ± SD	Control 24 h Mean ± SD	Δ Mean ± SD	*P*-value
Hemoglobin (g/dL)	13.24 ± 1.12	12.78 ± 1.18	−0.46 ± 0.40	.001	13.36 ± 1.15	12.33 ± 1.25	−1.03 ± 0.49	<.001
D-dimer (µg/mL)	0.27 ± 0.13	0.31 ± 0.15	+0.04 ± 0.07	.132	0.26 ± 0.12	0.49 ± 0.22	+0.23 ± 0.18	<.001
Fibrinogen (mg/dL)	2.90 ± 0.75	3.08 ± 0.70	+0.18 ± 0.34	.062	2.94 ± 0.71	3.44 ± 0.85	+0.50 ± 0.41	<.001

SD, standard deviation; TXA, tranexamic acid.

Fibrinogen concentrations showed a mild postoperative rise in both groups; however, the increase was milder in patients who received TXA (+0.18 ± 0.34 mg/dL, *P* = .062) compared with the control group (+0.50 ± 0.41 mg/dL, *P* < .001). Although the between-group difference in postoperative fibrinogen (3.08 ± 0.70 vs 3.44 ± 0.85 mg/dL) did not reach statistical significance (*P* = .064), the trend suggested less activation of the coagulation cascade in the TXA group.

### Safety and Adverse Events

No intraoperative or postoperative complications related to TXA administration were observed (Table 4). Specifically, there were no instances of thromboembolic events (deep vein thrombosis or pulmonary embolism), seizures, or allergic reactions in either group. There were no cases of skin-flap necrosis or delayed wound healing in either group. One patient in the control group developed a small hematoma that required aspiration, which resolved without any lasting effects. Another control patient experienced a small wound hematoma needing aspiration and was briefly readmitted, but no surgical reintervention was necessary. No reoperations or other adverse events occurred in patients who received TXA. Overall, the safety profile of intravenous TXA was excellent, with no adverse drug-related events recorded. All participants recovered without complications and were discharged within the planned postoperative period.

**Table 4.** Postoperative Complications and Adverse Events

Complication type	TXA group (*n* = 30)	Control group (*n* = 30)	*P*-value
Thromboembolic event (DVT/PE)	0 (0%)	0 (0%)	—
Seizures	0 (0%)	0 (0%)	—
Allergic or hypersensitivity reaction	0 (0%)	0 (0%)	—
Skin-flap necrosis or delayed wound healing	0 (0%)	0 (0%)	—
Wound hematoma requiring intervention	0 (0%)	1 (3.3%)	.312
Reoperation/readmission (30 days)	0 (0%)	1 (3.3%)	.312
Any adverse event	0 (0%)	1 (3.3%)	.312

DVT, deep vein thrombosis; PE, pulmonary embolism; TXA, tranexamic acid.

## DISCUSSION

This randomized, triple-blinded, placebo-controlled trial demonstrated that a single pre-incisional intravenous dose of TXA (10 mg/kg) significantly reduced total perioperative blood loss and attenuated postoperative fibrinolytic activity in women undergoing breast aesthetic surgery. Patients who received TXA exhibited ∼26% lower total blood loss compared with controls (221 vs 298 mL) and a markedly smaller increase in D-dimer concentration, confirming a systemic antifibrinolytic effect. Importantly, no thromboembolic, neurological, or allergic complications were observed, supporting the excellent safety profile of TXA in this population.

The present findings align with accumulating evidence from plastic and reconstructive surgery, indicating that TXA effectively reduces perioperative bleeding.^11^ Although most earlier studies have focused on abdominoplasty, rhinoplasty, or facelift procedures, the hemostatic mechanisms are similar across soft-tissue operations that involve extensive subcutaneous dissection and potential diffuse bleeding.^6,13,14^

In abdominoplasty, intravenous TXA at doses of 10 to 15 mg/kg has been shown to reduce total blood loss by 25% to 40% and decrease drain output without elevating the risk of thromboembolism. Similar findings have been reported in reduction mammaplasty, where both intravenous and topical administration of TXA resulted in significant decreases in postoperative drainage volumes and early bruising.^8,11,15^ Topical or irrigational TXA has become popular in aesthetic surgery; however, evidence indicates that its absorption and antifibrinolytic effects are less predictable than those of systemic administration. Comparative studies in abdominoplasty and breast reduction procedures have demonstrated that intravenous TXA achieves more consistent plasma concentrations and provides superior reductions in drainage volumes and hematoma formation compared with topical irrigation.^8,11,15^ Although topical TXA may offer localized benefit in high-risk bleeding areas, the systemic approach ensures uniform antifibrinolytic coverage during all phases of surgery, supporting our choice of intravenous administration in the present trial.

Administering the drug ∼10 min before incision ensures peak plasma levels coincide with the period of maximal intraoperative bleeding. Evidence from orthopedic and plastic surgery indicates that pre-incisional dosing provides more consistent hemostatic control and greater reductions in perioperative blood loss compared with intraoperative or postoperative administration.^8,11,16^ This rationale guided the dosing schedule used in our trial.

Our data support these findings in a more uniform group of aesthetic breast surgery patients, further confirming that the antifibrinolytic effectiveness of TXA applies to moderate-volume procedures with a relatively low baseline bleeding risk.^2,10,11^

The reduction in postoperative D-dimer levels observed in our study provides biochemical confirmation of TXA's mechanism of action. D-dimer is a sensitive marker of fibrinolysis and clot degradation; the more minor postoperative increase in the TXA group indicates inhibition of plasmin-mediated fibrin breakdown. Although fibrinogen levels rose modestly in both groups, the lower magnitude of increase after TXA may reflect reduced systemic inflammatory activation and improved hemostatic balance.^17^ These laboratory findings align with results from previous studies in orthopedic and trauma surgery, which have shown that TXA stabilizes fibrin clots by competitively inhibiting plasminogen binding to fibrin, thereby preserving clot integrity without excessive coagulation activation.^18^

From a clinical standpoint, the observed reduction in blood loss of ∼77 mL, although modest in absolute terms, is meaningful in aesthetic breast surgery, where even small decreases in oozing can translate into fewer postoperative hematomas, lower drain volumes, shorter drainage duration, and improved aesthetic outcomes.^12^ Minimizing subcutaneous bleeding also facilitates early recovery and may decrease tissue edema and ecchymosis, factors that influence patient satisfaction and perceived quality of surgical results.^19^

Importantly, all patients receiving TXA in our study maintained stable hemodynamic parameters, and none developed adverse drug reactions, seizures, or thromboembolic events. These data contribute to the growing body of evidence supporting TXA as a safe and effective adjunct in aesthetic surgery when administered at standard perioperative doses.^6,18^

The antifibrinolytic action of TXA is particularly relevant in breast aesthetic procedures, which often involve large areas of subcutaneous undermining and exposure of vascularized soft tissue.^2^ These conditions trigger local activation of plasminogen and fibrinolysis at the wound surface. By competitively inhibiting lysine-binding sites on plasminogen, TXA prevents the conversion of plasminogen to plasmin and subsequent fibrin degradation.^12^ The result is more stable clot formation and reduced capillary oozing, without interfering with primary hemostasis.^7,16^ Moreover, TXA may indirectly reduce the release of inflammatory mediators, which contributes to better postoperative tissue integrity.^20^

### Strengths and Limitations

The strengths of this trial include its randomized, triple-blind design, standardized anesthesia and surgical protocols, homogeneous patient population, and complete follow-up, which together minimize bias and enhance internal validity. The study was adequately powered to detect clinically relevant differences in total blood loss, and all analyses were performed according to the prespecified statistical plan. The absence of missing data or protocol violations strengthens the reliability of the results.

The present study has several limitations. First, its single-center design and relatively small sample size limit the generalizability of the findings. Nevertheless, our results are consistent with earlier randomized trials in aesthetic and reconstructive surgery, which also demonstrated significant reductions in postoperative bleeding with relatively small cohorts. Second, the study population consisted exclusively of low-risk patients (ASA I/II, BMI < 35 kg/m^2^), which limits applicability to higher-risk surgical populations. Third, blood loss was estimated using suction volumes and drain measurements rather than direct volumetric assessment or hemoglobin balance, an approach similar to that employed in previous plastic and orthopedic TXA studies. Finally, laboratory markers of fibrinolysis were measured only preoperatively and at 24 h postoperatively; additional time points could provide a more detailed understanding of TXA's pharmacodynamics. Future multicenter trials with larger, more diverse populations and extended biomarker profiling are warranted to confirm and expand these findings.

## CONCLUSIONS

Pre-incisional intravenous TXA safely reduces perioperative bleeding and fibrinolytic activity after reduction mammoplasty. Following this trial, intravenous TXA (10 mg/kg) has been incorporated into our institution's standard protocol for breast reduction and other aesthetic soft-tissue surgeries because of its demonstrated safety and efficacy.
